# Different routes and doses influence protection in pigs immunised with the naturally attenuated African swine fever virus isolate OURT88/3

**DOI:** 10.1016/j.antiviral.2016.11.021

**Published:** 2017-02

**Authors:** Pedro J. Sánchez-Cordón, Dave Chapman, Tamara Jabbar, Ana L. Reis, Lynnette Goatley, Christopher L. Netherton, Geraldine Taylor, Maria Montoya, Linda Dixon

**Affiliations:** The Pirbright Institute, Ash Road, Pirbright, Woking, Surrey, United Kingdom

**Keywords:** African swine fever, Pigs, Immunisation routes, Protection, Cytokines

## Abstract

This study compares different combinations of doses and routes of immunisation of pigs with low virulent African swine fever virus (ASFV) genotype I isolate OURT88/3, including the intramuscular and intranasal route, the latter not previously tested. Intranasal immunisations with low and moderate doses (10^3^ and 10^4^ TCID_50_) of OURT88/3 provided complete protection (100%) against challenge with virulent genotype I OURT88/1 isolate. Only mild and transient clinical reactions were observed in protected pigs. Transient moderate virus genome levels were detected in blood samples after challenge that decreased, but persisted until the end of the experiment in some animals. In contrast, pigs immunised intramuscularly with low and moderate doses (10^3^ and 10^4^ TCID_50_) displayed lower percentages of protection (50–66%), and low or undetectable levels of virus genome were detected in blood samples throughout the study. In addition, clinical courses observed in protected pigs were asymptomatic. In pigs that were not protected and developed acute ASF, an exacerbated increase of IL-10 sometimes accompanied by an increase of IFNγ was observed before euthanasia. These results showed that factors including delivery route and dose determine the outcome of immunisation with the naturally attenuated isolate OURT88/3.

## Highlights

• Protection induced by combinations of doses and routes of immunisation of pigs with ASFV isolate OUR T88/3 is described.
• Intranasal immunisations with low and moderate doses provided complete protection with persistent low virus genome levels.
• Intramuscular immunisations with low and moderate doses provided lower protection with undetectable virus genome levels.
• Increased IL-10 serum concentrations, sometimes together with increased IFNγ, was observed before death in not-protected pigs.
• Changes in serum concentrations of TNFα, IFNγ, IL-4 and IL-10 were not detected in none of immunised protected pigs.

## 1 Introduction

African swine fever (ASF) is a devastating disease of pigs caused by an enveloped DNA virus (Dixon et al., 2005), against which there is no available vaccine mainly due to the complexity of the virus, the technical difficulties involved in its development and to the fact that ASF has been traditionally considered an exotic disease in developed countries. ASFV genotype I isolates have been historically described as the main circulating isolates in the African continent, being a major limiting factor for pig production in most of sub-Saharan countries of Western and Central African (Boshoff et al., 2007, EFSA AHAW Panel, 2015). Genetically modified attenuated viruses, for which genes for virulence factors have been deleted, may provide safe vaccines against ASF (Lewis et al., 2000, O'Donnell et al., 2015, Reis et al., 2016). Despite some safety problems, live attenuated vaccines have demonstrated a high effectiveness against experimental infections with homologous (Leitão et al., 2001, Boinas et al., 2004, Oura et al., 2005, King et al., 2011, Lacasta et al., 2015), and occasionally heterologous (King et al., 2011, Mur et al., 2013), virulent isolates of ASFV. Like other viruses, ASFV has developed strategies to manipulate essential cytokines for the regulation of the immune response and the outcome of the infection (Dixon et al., 2004, Correia et al., 2013). Different studies have also suggested the potential role of IFNγ producing cells in protection against ASFV (Alonso et al., 1997, King et al., 2011, Argilaguet et al., 2012, Argilaguet et al., 2013). However, the role of cytokines in regulating the protective immune response *in vivo* has been poorly studied, and mainly focused on infections with highly virulent (Salguero et al., 2002, Salguero et al., 2005, Sánchez-Cordón et al., 2008, Zakaryan et al., 2015) rather than low-virulent ASFV isolates.

Protection induced by oronasal immunizations of pigs with low virulence ASFV isolates, especially protection mechanisms induced by the naturally low-virulent NH/P68 isolate, have been previously described *in vivo* by using inbred and outbred pigs (Scholl et al., 1989, Martins et al., 1993, Leitão et al., 2001). The present study extends previous studies on protection induced by intramuscular administration of OURT88/3 (Boinas et al., 2004, Oura et al., 2005, King et al., 2011, Abrams et al., 2013). Here, different combinations of doses (10^3^, 10^4^ and 10^5^ TCID_50_) and routes of immunisation of OURT88/3, including the intranasal route which had not been previously tested, were evaluated. The results obtained provide valuable information about percentages of protection, appearance of clinical signs along with differences in anti-ASFV antibody response and in the serum concentration of immunoregulatory cytokines between protected and not-protected immunised pigs.

## 2 Material and methods

### 2.1 Cells and viruses

Both low virulence, non-haemabsorbing genotype I ASFV isolate OURT88/3 and virulent haemabsorbing genotype I isolate OURT88/1 (Boinas et al., 2004) were grown in primary macrophage cultures derived from bone marrow. Virus titration was carried out as previously reported (Guinat et al., 2014). Titres of virus were determined by identification of viral protein VP30 (mouse monoclonal IgG1 antibody, clone C18, Pirbright Institute) using immunofluorescence. Results are presented as the amount of virus infecting 50% of the macrophage cultures (TCID_50_/ml).

### 2.2 Experimental design

Experiments were conducted in BSL-3 facilities at CReSA (Barcelona, Spain) according to regulated procedures from the Animals (Scientific Procedures) Act 1986. Groups of six Large White and Pietrain crossbred male piglets, eight-week-old, vaccinated against Porcine Circovirus type 2 (PCV2) and Mycoplasma hyopneumoniae from a high health status herd tested negative for Porcine Respiratory and Reproductive syndrome (PRRS) and Aujeszky's disease were used (Table 1). Groups of pigs were immunised intramuscularly (IM) with 1 ml containing 10^3^ (group A), 10^4^ (group C) and 10^5^ (group E) TCID_50_/ml or intranasally (IN) with 1 ml per nostril containing 10^3^ (group B), 10^4^ (group D) and 10^5^ (group F) TCID_50_/ml of low virulent ASFV isolate OURT88/3. Mucosal atomization devices (MAD300, DS Medical) capable of generating particles of 30–100 microns size were used. Three weeks later all immunised groups, together with a control group (group G) containing three non-immunised pigs, were challenged intramuscularly with 1 ml containing 10^4^ TCID_50_/ml of the genotype I virulent ASFV isolate OURT88/1.

### 2.3 Sampling, clinical and post-mortem examination

Immunisation day was defined as day 0 (0 dpi). Rectal temperatures and clinical signs were monitored daily as described (King et al., 2011). EDTA blood and serum samples were collected from all pigs prior to virus immunisation (0 dpi), after immunisation (at 3, 5, 7, 14 and 21 dpi) and post-challenge (at 3, 5, 7, 14 and 19 dpc). Samples were frozen at −80 °C.

During the experiment, unprotected pigs were euthanized at different time-points after reaching a specified endpoint, while all protected pigs were euthanized at 19 dpc. Gross lesions were evaluated in accordance with standardized protocols (Galindo-Cardiel et al., 2013). Tissue samples from spleen, tonsil, lung, submandibular, retropharyngeal and gastrohepatic lymph nodes were collected from two protected immunised pigs belonging to each of the experimental groups (pigs A1, A2, B1, B2, C1, C2, D1, D2, E1, E4, F1 and F2), from some immunised not-protected pigs randomly selected (E2, E3, E5, E6, F3) and from one non-immunised control pig (pig G3). Tissue samples were frozen at −80 °C.

### 2.4 ASFV detection and immune response evaluation

Blood and tissues samples were analysed for ASFV genome detection by quantitative PCR (qPCR) (King et al., 2003). Total genome copies in blood and tissue samples were expressed per millilitre (/mL) or per gram (/g) respectively. Levels of infectious virus in blood samples were also estimated by virus titration.

Serum samples were assayed by commercial ELISA kits (Blocking ELISA) for detection of ASFV-specific antibodies against VP72 (INGEZIM PPA Compac, Ingenasa) or porcine immunoregulatory cytokines (TNFα, IFNγ, IL-4 and IL-10) (R&D Systems).

### 2.5 Statistical analysis

Data from rectal temperatures, clinical scores, anti-ASFV specific antibody levels and cytokine concentrations were statistically analysed using GraphPad Prism Version 6.0 (GraphPad Software).

## 3 Results

### 3.1 Not-vaccinated control pigs developed acute ASF

**Supplementary Fig. 1:**
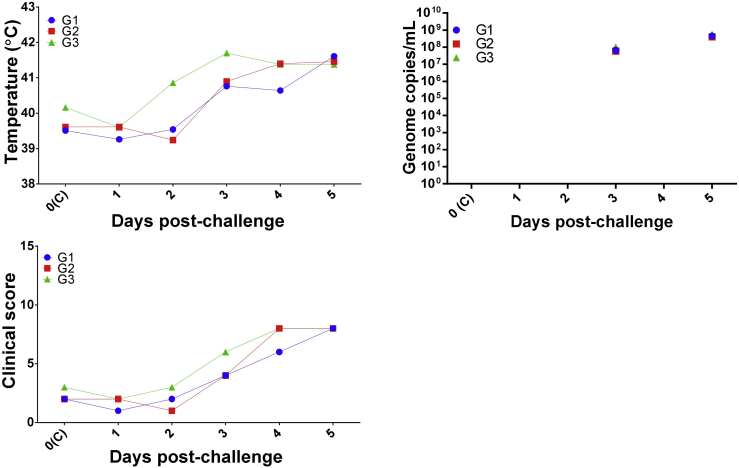
Clinical evaluation and viremia in unvaccinated control pigs. Rectal temperatures, clinical scores and viremia (*y*-axis) were assessed at different days after challenge (C) of pigs (*x-*axis). Pigs were challenged intramuscularly with 1 ml containing 10^4^ TCID_50_/ml of the genotype I virulent ASFV isolate OURT88/1.

All control pigs were euthanized at 5 dpc showing viremia (up to 5.6 × 10^8^ copies/mL), clinical signs and gross lesions characteristic of acute ASF (Galindo-Cardiel et al., 2013, Sánchez-Vizcaíno et al., 2015) (Supplementary Fig. 1). Infectious ASFV was also confirmed in blood by virus titration (data not shown). Levels of virus genome in tissue samples from control pig G3 ranged between 4.66 × 10^7^ copies/g in tonsil to 2.41 × 10^8^ copies/g in spleen.

### 3.2 Intranasal immunizations with low and moderate doses provided complete protection with minimum clinical reactions while high doses induced chronic forms of ASF

**Fig. 1:**
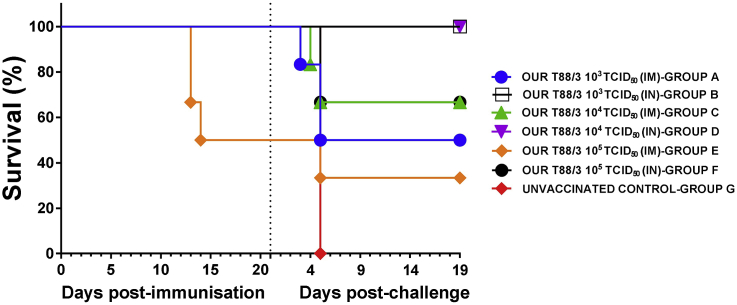
Percentage of surviving animals. Groups of pigs (n = 6) were immunised intramuscularly (IM) or intranasally (IN) with different doses (10^3^, 10^4^ and 10^5^ TCID_50_/ml) of low virulent ASFV isolate OURT88/3 genotype I. Three weeks later all immunised groups, together with a control group of non-immunised pigs (n = 3), were challenged intramuscularly with 10^4^ TCID_50_/ml of virulent ASFV isolate OURT88/1 genotype I. Days post-immunisation or post-challenge (*x-*axis); Percent survival (*y*-axis).

**Fig. 2:**
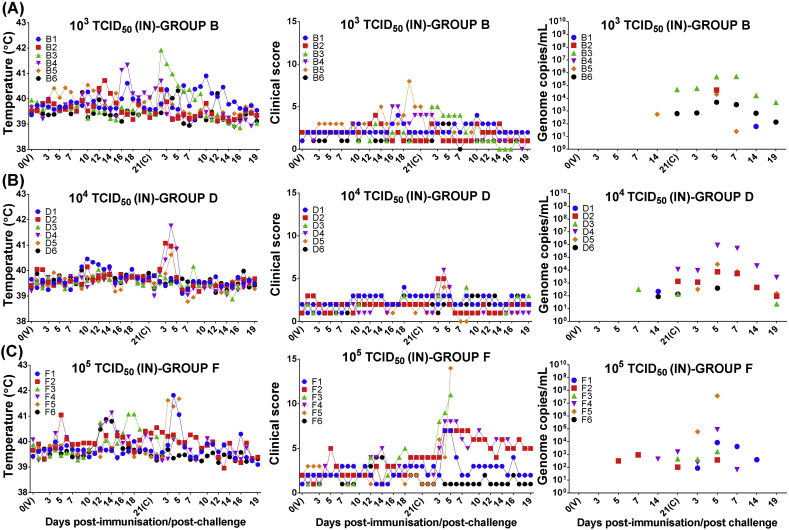
Clinical evaluation and viremia in pigs immunised by intranasal route. Rectal temperatures, clinical scores and viremia (*y*-axis) were assessed at different days after intranasal immunisation (V) and challenge (C) of pigs (*x-*axis). Pigs were immunised intranasally with different doses of low virulent ASFV isolate OURT88/3: 10^3^ TCID_50_/ml (panel A); 10^4^ TCID_50_/ml (panel B); 10^5^ TCID_50_/ml (panel C). Viral genome copies in blood samples was determined by qPCR and expressed as total genome copies per millilitre (/mL).

All of the pigs immunised intranasally with 10^3^ (group B) and 10^4^ (group D) TCID_50_ of OURT88/3 were protected, whereas just 4 out of 6 pigs immunised intranasally with 10^5^ TCID_50_ (group F) survived (Fig. 1). In some of the protected pigs from groups B and D, transient and intermittent mild/moderate joint swelling was the only remarkable clinical sign observed both before and after challenge. In addition, pig B3 and three pigs from group D (pigs D2, D4 and D5) also showed a short transient clinical picture after challenge (2–5 dpc) with temperatures up to 41.9 °C and non-specific clinical signs (inappetence and apathy) (Fig. 2a and b). Such clinical signs were associated with a transient increase of viremia (up to 5.1 × 10^5^ copies/mL for pig B3 and 8.7 × 10^5^ copies/mL for pig D4) that decreased from 7 dpc onwards. Viremia in pigs from groups B and D was very low or not-detected at the end of the experiment (19 dpc). These blood samples were also negative by virus titration (data not shown). In addition, all tissue samples collected post-mortem from pigs B1, B2, D1 and D2 were negative for ASFV when analysed by qPCR (data not shown).

Two pigs from group F (IN, 10^5^ TCID_50_) were euthanized at 5 dpc (Fig. 2c). Pig F5 showed characteristic signs of acute ASF (41.7 °C, skin erythema, hemorrhagic internal lesions, high viremia up to 3.7 × 10^7^ copies/mL), while pig F3 developed signs typical of chronic forms of ASF (Sánchez-Vizcaíno et al., 2015). These included temperature of 40.0 °C, low viremia below 1.7 × 10^3^ copies/mL with negative results in virus titration, severe joint swelling, skin erosions in the nose containing up to 2 × 10^8^ genome copies/g and nonspecific internal lesions in the lungs.

Protected pigs in group F (F1, F2, F4 and F6) also displayed signs of chronic ASF including joint swelling, laboured breathing, conjunctivitis, skin erosions/ulcers in nose, flanks and limbs and cardiorespiratory lesions consistent with the presence of secondary bacterial infections. After challenge, only pig F1 (Fig. 2c) showed transient high temperature along with mild viremia (8.4 × 10^3^ copies/mL) which decreased from 7 dpc onwards, while in pigs F2, F4 and F6 viremia was very low or not detected. At termination (19 dpc), none of the protected pigs had detectable virus genome in blood and all blood samples were also negative by virus titration (data not shown). ASFV was also not detected by qPCR in tissue samples taken from pigs F1 and F2 (data not shown).

### 3.3 Intramuscular immunisations induced lower protection with low or undetectable viral loads in blood and asymptomatic clinical courses in protected pigs

**Fig. 3:**
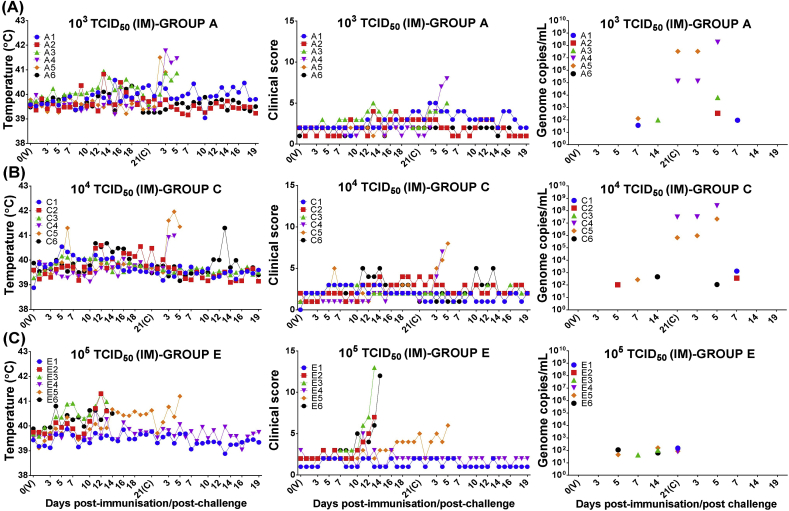
Clinical evaluation and viremia in pigs immunised by intramuscular route. Rectal temperatures, clinical scores and viremia (*y*-axis) were assessed at different days after intramuscular immunisation (V) and challenge (C) of pigs (*x-*axis). Pigs were immunised intramuscularly with different doses of low virulent ASFV isolate OURT88/3: 10^3^ TCID_50_/ml (panel A); 10^4^ TCID_50_/ml (panel B); 10^5^ TCID_50_/ml (panel C). Viral genome copies in blood samples was determined by qPCR and expressed as total genome copies per millilitre (/mL).

Survival percentages in pigs immunised intramuscularly were 50% (10^3^ TCID_50_, group A) and 66% (10^4^ TCID_50_, group C) respectively (Fig. 1). Pigs in groups A (A3, A4 and A5) and C (C4 and C5) were found dead or euthanized between 4 and 5 dpc after developing acute ASF with high temperatures, high viremia levels up to 2.4 × 10^8^ copies/mL and haemorrhagic internal lesions (Fig. 3a and b).

In contrast, three pigs in group E (E2, E3, and E6) were euthanized before challenge between 13 and 14 dpi (Fig. 3c) with temperatures ranging from 40.5 to 41.3 °C and nonspecific but high clinical scores. These animals also showed nonspecific internal chronic lesions mainly affecting small areas of pulmonary cranial lobes which might have been caused by previous infections with bacteria such as Mycoplasma hyopneumoniae, although other aetiological agents should not be ruled out. Viremia was either very low or undetectable by qPCR and negative by virus titration. Tissue samples also displayed low numbers of viral genome copies (<10^3^ copies/g), except in retropharyngeal lymph nodes of pig E3 (5.4 × 10^4^ copies/g). Virology results were not consistent with acute or subacute forms of ASF, however the short clinical courses observed (13–14 days) along with the nonspecific clinical signs and lesions also ruled out chronic ASF in these pigs.

However, pig E5 was euthanized at 5 dpc with signs of chronic ASF including joint swelling, skin erosions in the nose and around the joints along with nonspecific internal gross lesions. Viremia was less than 10^3^ copies/mL or undetectable, however high numbers of viral genome copies were detected in skin lesions from the nose (3.5 × 10^8^ genome copies/g), skin lesions around swollen joints (1.6 × 10^5^ genome copies/g) and submandibular lymph nodes (1.06 × 10^6^ genome copies/g).

Finally, transient mild joint swelling was the only clinical sign observed in most of the protected pigs immunised intramuscularly (A1, A2, A6, C1, C2, C3, C6, E1 and E4), which generally displayed lower genome copy levels than pigs protected by intranasal immunisation. Virus genome was not detected from 14 dpc onwards and virus titration was negative in blood samples taken at termination. All tissue samples analysed from pigs A1, A2, C1, C2, E1 and E4 were also negative by qPCR.

### 3.4 Not-protected immunised pigs with the highest peaks of viremia showed irregular anti-ASFV antibody response

**Fig. 4:**
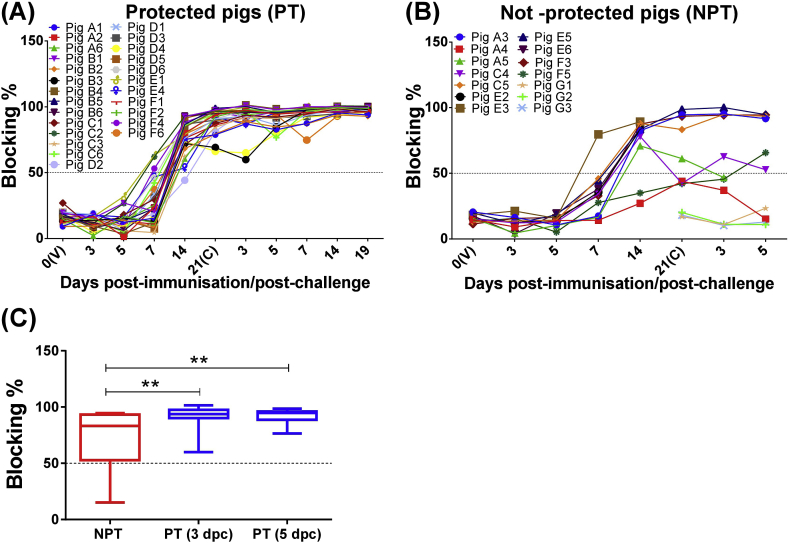
Anti-ASFV VP72 antibody response. Individual kinetic of ASFV-specific antibodies in serum samples from protected pigs (PT; panel A) and not-protected pigs (NPT; panel B) at different days following immunisation (V) and challenge (C) (*x-*axis). Panel C shows mean and SD of blocking percentages (*y*-axis) detected in serum samples taken just before euthanasia in the group of pigs not-protected (NPT) between 3 and 5 dpc and samples taken at 3 and 5 dpc in the group of protected pigs (PT). Statistical analysis was carried out using a Mann–Whitney *U* test for non-parametric distributions. Asterisks indicate statistically significant differences between groups of pigs (**P < 0.01). Levels of anti-VP72 antibodies were measured by blocking ELISA assay. Serum samples with a blocking percentage ≥50% were considered positive (the cut off is represented as dashed line parallel to the *x-*axis).

All immunised pigs (except pig A4) developed a positive antibody response against VP72 which was not detected in control pigs (G1, G2 and G3). In most of protected pigs, the presence of antibodies remained high after 14 dpi (Fig. 4a), suggesting a saturation of blocking ELISA as consequence of high concentrations of ASFV-specific antibodies.

However, variation in antibody levels were observed among not-protected pigs (Fig. 4b). So, while antibody response in not-protected pigs A3, C5, E5 and F3 was similar to observed in protected pigs, fluctuations together with a lower presence of antibodies were observed in pigs A4 (negative antibody response), A5, C4 and F5 throughout the experiment. The latter also displayed the highest peaks of viremia.

Regarding the presence of ASFV-specific antibodies against VP72, significant statistical differences (*P* < 0.01) were detected among serum samples taken just before euthanasia in the group of pigs not-protected (between 3 and 5 dpc) and samples taken at 3 and 5 dpc in the group of protected pigs (Fig. 4c).

### 3.5 Protected pigs did not display changes in serum cytokine levels, whereas an increase of IL-10 and IFNγ appeared post challenge in non-protected pigs with acute ASF

Data on cytokine levels from pigs euthanized during the experiment were excluded from comparative statistical analysis in those cases in which acute ASF was not confirmed (pigs E2, E3, E5, E6 and F3).

**Supplementary Fig. 2:**
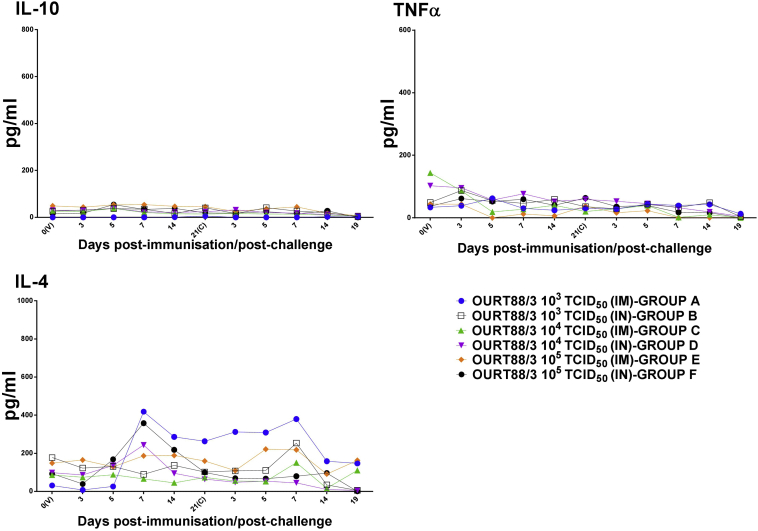
Comparative study of the mean concentrations of cytokines (IL-10, TNFα and IL-4) detected in serum samples of protected pigs immunised by different doses and routes with low virulent ASFV isolate OURT88/3 genotype I and challenged intramuscularly with 10^4^ TCID_50_/ml of virulent ASFV isolate OURT88/1 genotype I. Days post-immunisation (V) or post-challenge (C) (*x-*axis); Mean concentratrions of cytokines expressed as pg/ml (*y*-axis). A graph of serum levels of IFNγ in protected pigs has not been included because in none of protected pigs serum concentration of IFNγ were detected neither after immunisation, nor after challenge. Statistical analysis was carried out using a two-way ANOVA with Bonferroni post-test.

**Fig. 5:**
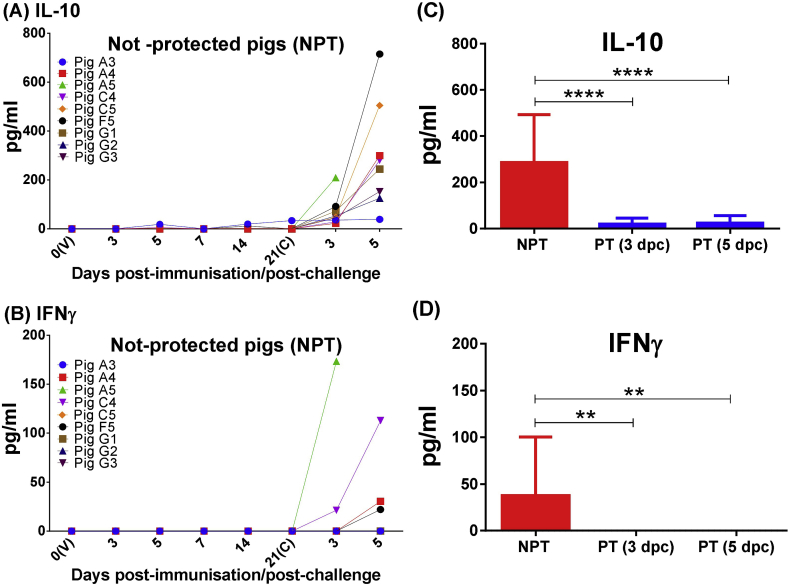
Changes in serum concentrations of IL-10 and IFNγ. Individual kinetic of IL-10 (panel A) and IFNγ (panel B) in serum samples from not-protected pigs (including immunised pigs and non-immunised control pigs) at different days following immunisation (V) and challenge (C) (*x-*axis); Serum concentration (mean and SD) of IL-10 (panel C) and IFNγ (panel D) detected in serum samples taken just before euthanasia in the group of pigs not-protected (including immunised pigs and non-immunised control pigs euthanized between 3 and 5 dpc) and samples taken at 3 and 5 dpc in the group of protected pigs. Statistical analysis was carried out using a Mann–Whitney *U* test for non-parametric distributions. Asterisks indicate statistically significant differences between groups of pigs (**P < 0.01; ****P < 0.0001). Data on cytokine levels from pigs euthanized during the experiment were excluded from comparative statistical analysis in those cases in which acute ASF was not confirmed (pigs E2, E3, E5, E6 and F3). Cytokines levels in serum samples were assayed by commercial ELISA kits and results were expressed as pg/ml. Protected pigs (PT); Not-protected pigs (NPT).

There were no significant changes in the serum concentrations of TNFα, IL-4, IL-10 and IFNγ among groups of protected pigs after vaccination or challenge at the time points tested (Supplementary Fig. 2). However, changes in serum levels of IL-10 and IFNγ were detected post-challenge in pigs that suffered acute ASF (both control and vaccinated). Except for pig A3, all non-protected pigs displayed an increase of IL-10 serum concentrations just before euthanasia (Fig. 5a). Levels were especially high in pigs A4, A5, C4, C5 and F5 that also displayed the highest viremia levels. Curiously, non-protected pigs in which acute ASF was not confirmed, and where viremia was either not detected, or detected at low levels (pigs E2, E3, E5, E6 and F3), did not show an increase of IL-10 before death (data not show). Regarding IFNγ (Fig. 5b), a high increase in serum concentrations was detected just before euthanasia in non-protected pigs that showed the highest levels of viremia (A4, A5, C4 and F5). These changes were not observed in any of the non-immunised control pigs (G1, G2 or G3).

**Fig. 6:**
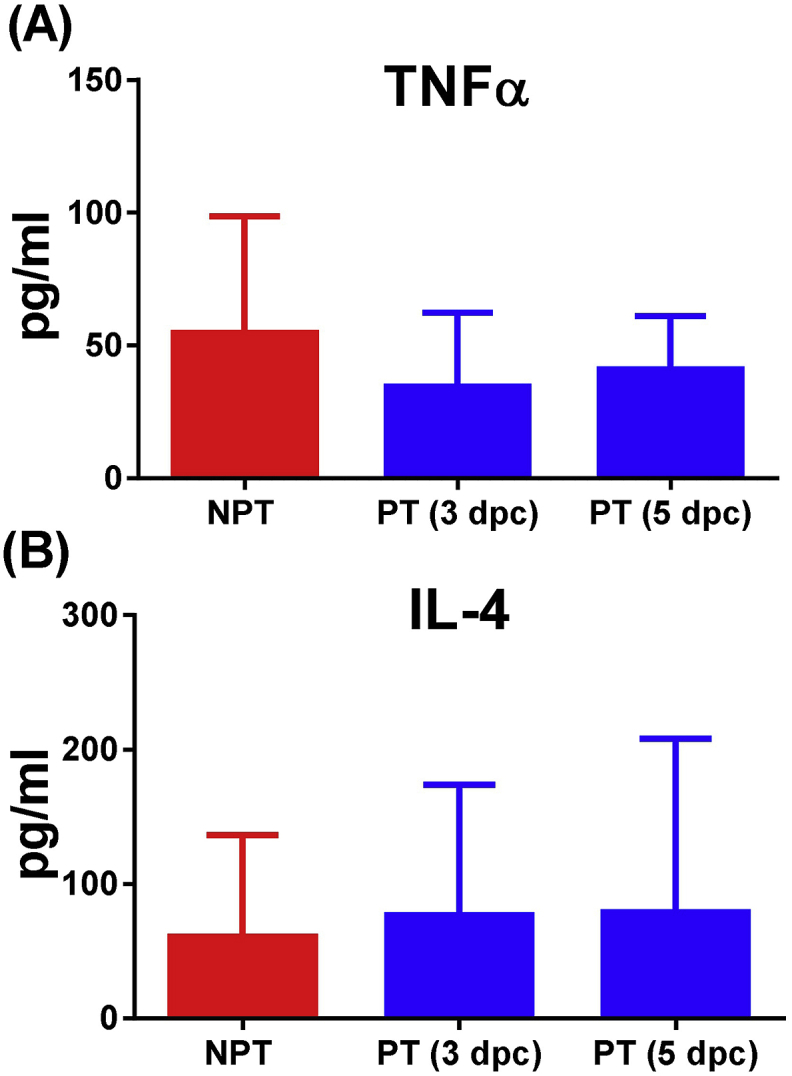
Changes in serum concentrations of TNFα and IL-4. Serum concentration (mean and SD) of TNFα (panel A) and IL-4 (panel B) detected in serum samples taken just before euthanasia in the group of pigs not-protected (including immunised pigs and non-immunised control pigs euthanized between 3 and 5 dpc) and samples taken at 3 and 5 dpc in the group of protected pigs. Statistical analysis was carried out using a Mann–Whitney *U* test for non-parametric distributions. Significant differences were not detected among groups. Data on cytokine levels from pigs euthanized during the experiment were excluded from comparative statistical analysis in those cases in which acute ASF was not confirmed (pigs E2, E3, E5, E6 and F3). Cytokines levels in serum samples were assayed by commercial ELISA kits and results were expressed as pg/ml. Protected pigs (PT); Not-protected pigs (NPT).

Statistical analysis showed significant differences in serum concentrations of IL-10 (P < 0.0001) and IFNγ (P < 0.01) among samples taken just before euthanasia in the group of pigs not-protected (between 3 and 5 dpc) and samples taken at 3 and 5 dpc in the group of protected pigs (Fig. 5c,d). Such differences were not observed in serum concentrations of TNFα and IL-4 (Fig. 6).

Finally, pigs E2, E3 and E6, which were immunised intramuscularly (10^5^ TCID_50_) and euthanized before challenge (between 13 and 14 dpi), showed very high initial concentrations of IL-10 that persisted until before euthanasia (data not shown).

## 4 Discussion

Pigs inoculated intramuscularly with 10^4^ TCID_50_ were protected to a similar degree (66%) as previously described (Boinas et al., 2004, Oura et al., 2005, King et al., 2011, Abrams et al., 2013). However, the level of protection induced when pigs were immunised intramuscularly with 10^3^ and 10^5^ TCID_50_ was lower (50 and 33% respectively). Interestingly pigs E2, E3 and E6 immunised intramuscularly with 10^5^ TCID_50_ were euthanized 13–14 days after vaccination. The lack of lesions consistent with ASF coupled with the very low or undetectable levels of viremia ruled out the existence of acute or subacute forms of ASF in such pigs. These pigs had high serum concentrations of IL-10 prior to immunisation which persisted throughout the experiment until the day of euthanasia. The presence of high concentrations of this cytokine, involved in inflammatory and immune response modulation (Moore et al., 1993, Moore et al., 2001), together with non-specific clinical signs suggested that these animals might have suffered from an undetermined subclinical infection. In a recent experiment, pigs immunised intramuscularly with a high dose of OURT88/3 (10^5^ TCID_50_) and challenged with virulent genotype I isolate showed 100% protection, along with mild clinical signs and lesions (joint swelling and skin erosions) similar to those described in the present study (unpublished data). So, these results would rule out the high dose of OURT88/3 immunised intramuscularly in this experiment as the main cause of the unknown subclinical infections described.

In agreement with previous studies with low virulent ASFV isolate (NH/P68) (Leitão et al., 2001), intranasal immunisations with OURT88/3 induced higher levels of protection than intramuscular immunisation. The different doses administered intranasally gave rise to two clinical groups: protected pigs that developed transient clinical reactions (immunised with 10^3^ and 10^4^ TCID_50_, 100% of protection) and protected pigs which developed chronic forms of ASFV (immunised 10^5^ TCID_50_, 66% of protection). Most of the pigs that were protected by intranasal immunisation had recurrent viremia from 14 dpi. In addition, most of the protected pigs from the groups that were immunised with 10^3^ and 10^4^ TCID_50_ by the intranasal route, had persistent viremia until the end of the study (19 dpc). Interestingly, intramuscular immunisation did not lead to protected pigs suffering signs of chronic ASF. Viremia was not detected after 7 dpc and in most of the pigs, any viremia observed before then was less than 10^3^ copies/mL. These results contrast with those previously described where intranasal inoculation with NH/P68 led to fewer animals developing chronic ASF and lower viremia levels than pigs that had been inoculated intramuscularly (Leitão et al., 2001). In addition, a correlation between viremia late after immunisation with NH/P68 (after 14 days p.i) and the appearance of pigs with chronic ASF has been suggested (Leitão et al., 2001, Gallardo et al., 2015). Such correlation was not observed in protected pigs immunised intranasally with OURT88/3 where late viremia was described in pigs without chronic ASF. However, there was great similarity as regards the absence of clinical signs and low levels of viremia between protected pigs immunised intramuscularly in our experiments and protected pigs immunised by intramuscular or intranasal route with NH/P68 (Leitão et al., 2001, Gallardo et al., 2015).

Despite the complexity of standardizing a dose for intranasal immunisation, this route is presented as a feasible alternative for the protection of pigs against ASFV. Little is known about the immunological mechanisms underlying the induction of protection or about the possible role played by the mucosal immune system. It is well documented that the main function of mucosa-associated lymphoid tissue (MALT) is the selective absorption and uptake of antigens which may induce effective and long lasting local immunity by interacting with mucosal-associated lymphoid tissues or protective systemic immunity upon reaching other lymphoid organs such as spleen or lymph nodes (Almeida and Alpar, 1996, Zaman et al., 2013). However such local immune mechanisms, and its systemic consequences, have not been elucidated so far in immunised protected pigs against ASFV.

However, for vaccine development, safety is a key criterion. So, although pigs immunised with low and moderate doses by intramuscular route displayed lower protection, the low or non-existent viral loads together with the asymptomatic clinical courses observed in protected pigs, similar to those described in previous studies with OURT88/3 (Boinas et al., 2004, Oura et al., 2005, King et al., 2011, Abrams et al., 2013), suggested this route as the most feasible and safe for immunisations against ASFV.

High levels of anti-ASFV specific antibodies have been associated with chronic ASF while low levels have been described in asymptomatic pigs after intranasal and intramuscular immunisations with low virulent ASFV isolate (NH/P68) (Leitão et al., 2001, Gallardo et al., 2015). Such results contrast with those obtained in our study where, regardless of doses and routes of immunisations, a strong antibody response against VP72 structural protein was observed in all protected pigs from 14 dpi onwards, including those with and without signs of chronic ASF. A similar antibody response was also observed in 50% of the immunised pigs that were not protected. However, the four pigs that displayed higher peak viremia (A4, A5, C4 and F5), showed a weaker and more variable antibody response. Although this anomalous behaviour may suggest a potential role of humoral immune response in protection against ASFV (Escribano et al., 2013), a plausible explanation might also be that high levels of virus, as detected in these pigs, might interfere with the ELISA test, since antibodies may bind to viral particles which would mask their detection. Other studies where pigs were immunised with OURT88/3 (Oura et al., 2005) suggested that although anti-ASFV antibodies were not enough to protect pigs, their role should not be completely ruled out. As for cellular immune responses, subsets of NK cells and CD8^+^ T-cells have been indicated to be relevant for protection induced by some live attenuated vaccine candidates (Scholl et al., 1989, Leitão et al., 2001, Oura et al., 2005, Lacasta et al., 2015) and DNA vaccines (Argilaguet et al., 2012, Lacasta et al., 2014). More specifically, in protected pigs immunised by oronasal route with ASFV NH/P68 isolate, cytotoxic activity was mediated by CD8^+^ cells which lysed macrophages infected with homologous isolates under restriction of class I swine leukocyte antigen (SLA) (Martins et al., 1993). Further studies are required to establish the true relevance of antibody-mediated and cell-mediated protection.

Regarding the role played by cytokines in the mechanisms of protection, conversely to what other studies suggested (Lacasta et al., 2015), IL-10 did not seem to contribute to controlling ASFV replication or avoiding harmful inflammation. Instead, and given the ability of IL-10 to impair or counteract the innate and adaptive immune response (Moore et al., 1993, Moore et al., 2001), the high concentrations detected after challenge in most of the immunised non-protected pigs might be linked to a failure of protective immune responses that might compromise the pigs' survival. On the contrary, the absence of changes in IL-10 concentrations in protected pigs, might contribute to the activation of protective immune mechanisms able to control ASFV.

Finally, those pigs euthanized after challenge with the highest levels of viremia and IL-10 (pigs A4, A5, C4 and F5, but not C5), also displayed a high increase of IFNγ in serum before death that may be interpreted as a failed last attempt of immune system for controlling last stages of infection. Such ineffectiveness of IFNγ for inhibiting ASFV replication has been previously suggested in pigs experimentally infected with a highly virulent isolate, where high increases of IFNγ quantified by ELISA were detected just before death (Karalyan et al., 2012). Elevated IFN-γ levels have been associated with specific immune responses; however, the data presented here suggested that IFN-γ levels in serum at the last stages of ASFV might be the result of an adverse pathological condition without protection functions. Further experiments are required to elucidate the role of IFN-γ at different stages and tissues (systemic versus local secretion) after ASFV infection.

In conclusion, although intranasal inoculations of pigs with low-moderate doses of OURT88/3 provided a complete protection against homologous virulent ASFV, intramuscular immunisation with similar doses appeared as the most feasible and safe route despite showing lower percentages of protection. An exacerbated increase of serum IL-10 sometimes accompanied by an increase of IFNγ, was observed before euthanasia in most of not-protected pigs that suffered acute ASF, changes that might be associated with an uncontrolled cytokine response induced by the intense replication of ASFV.

## Conflict of interest

The authors declare they have not conflict of interest.

## Figures and Tables

**Table 1:** Experimental design.

Experimental group	No. of pigs	Immunisation route	Immunisation dose of OUR T88/3 (TCID_50_/ml)	Challenge by IM inoculation of OUR T88/1 (TCID_50_/ml)
A	n = 6	IM	10^3^	10^4^
B	n = 6	IN	10^3^	10^4^
C	n = 6	IM	10^4^	10^4^
D	n = 6	IN	10^4^	10^4^
E	n = 6	IM	10^5^	10^4^
F	n = 6	IN	10^5^	10^4^
G (Control)	n = 3	–	–	10^4^

IN, Intranasal immunisation; IM, intramuscular immunisation.

## Glossary

*ASF*: African swine fever
*ASFV*: African swien fever virus
*TCID*_*50*_: tissue culture infective dose
*BSL-3*: biosafety level 3
*EDTA*: ethylenediaminitetra-acetic acid
*ELISA*: enzyme-linked immunoabsorbent assay
